# Unraveling the Intertwined Networks of Potato Tuberization: From Epitranscriptomic Signaling to Metabolic Reprogramming

**DOI:** 10.3390/ijms27167149

**Published:** 2026-08-10

**Authors:** Dong Wang, Wenrui Zhao, Mingshou Fan, Ziyi Zhang

**Affiliations:** 1 Inner Mongolia Key Laboratory of Plants Adversity Adaptation and Genetic Improvement in Cold and Arid Regions, College of Life Sciences, Inner Mongolia Agricultural University, Hohhot 010018, China; wd0127@126.com (D.W.); 13614748377@139.com (W.Z.); 2 College of Agriculture, Inner Mongolia Agricultural University, Hohhot 010018, China; fmswh@126.com

**Keywords:** *Solanum tuberosum*, tuberization, StSP6A, epitranscriptomics, hormonal crosstalk, source-sink transition, diploid breeding, m^6^A methylation

## Abstract

Potato (*Solanum tuberosum* L.) tuberization is a complex developmental transition in which a subterranean stolon is converted into a starch-accumulating tuber. This process is governed by long-distance systemic signals, local hormonal dynamics, and extensive metabolic reprogramming. Recent advances have extended our understanding beyond the classical photoperiodic model, revealing the importance of chromatin remodeling, mRNA N^6^-methyladenosine (m^6^A) epitranscriptomic modifications, and a developmentally regulated shift in phloem unloading pathways. Here, we synthesize these multilayered regulatory networks into an integrated framework. We also examine the thermosensitivity of the mobile tuberigen signal under climate warming, assess the genetic challenges imposed by autotetraploidy and gene dosage, and discuss recent progress in diploid hybrid breeding strategies. In addition, we highlight emerging evidence for the roles of mechanoperception and rhizosphere microbiota as previously overlooked modulators of tuberization. Finally, we outline how these fundamental insights can be translated into breeding pipelines, with a focus on genome editing of cis-regulatory elements and the design of F1 hybrid cultivars. This review provides a conceptual roadmap for engineering climate-resilient, high-yielding potato cultivars to support global food security.

## 1. Introduction

Potato (*Solanum tuberosum* L.) is the world’s most important tuber crop and a staple food of global significance, providing essential carbohydrates, vitamins, and minerals to billions of people worldwide [1]. Unlike major cereal crops, which store starches in aerial seeds, potato derives its economic yield entirely from its underground tubers [1]. Morphologically, tuberization represents a profound post-embryonic developmental switch. It requires an elongated, plagiotropic, and geotropically sensitive underground stem (the stolon) to cease longitudinal extension and initiate rapid periclinal cell division and radial expansion at its subapical region [2].

For several decades, the molecular investigation of tuberization heavily paralleled the paradigms established for floral induction [3,4]. The landmark identification of Solanum tuberosum SELF-PRUNING 6A (StSP6A) as the long-sought, mobile “tuberigen” reinforced the conceptual parallel between flowering and tuberization [5]. Just as FLOWERING LOCUS T (FT) acts as a systemic florigen in Arabidopsis, the leaf-synthesized StSP6A protein undergoes long-distance translocation through the phloem to induce underground organ modification [6].

However, unlike the determination of a floral meristem, tuberization is a more complex process that requires complete morphological, cell-biological, and metabolic reprogramming of an entire storage tissue [7,8]. The linear pathways that dominated early research are proving insufficient to explain how the potato plant coordinates fluctuating environmental inputs with massive carbohydrate partition [9].

This review moves beyond a historical chronology of gene discovery. Instead, we synthesize a multi-layered regulatory network, integrating recent breakthroughs in chromatin accessibility, post-transcriptional RNA epitranscriptomics, localized hormonal antagonism, and vascular sucrose transport thermodynamics. Finally, we bridge the gap between basic molecular models and translational agronomy, offering an evolutionary and biotechnological perspective on how to engineer the potato crops of the future. To provide a clear conceptual roadmap for the reader, this review is structured around the following scope and central hypothesis.

**Figure 1:**
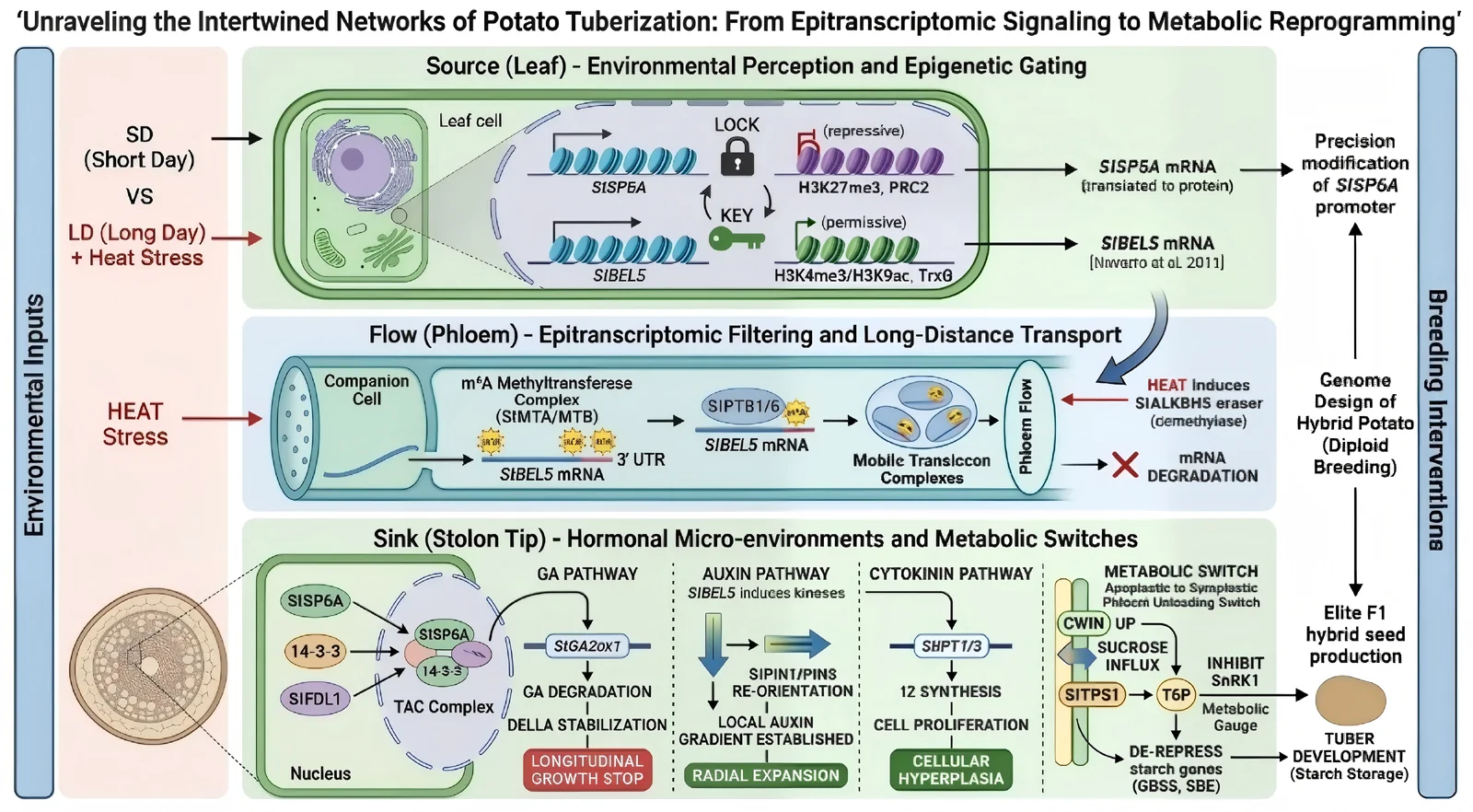
Integrated multi-layered regulatory network of potato tuberization spanning photoperiodic perception, phloem translocation, and stolon morphogenesis.

To provide a conceptual roadmap for the intricate interactions discussed in the following sections, we present a comprehensive schematic integrating the three major physiological compartments—source leaf, phloem stream, and stolon tip—in Figure 1.

The schematic is organized into three interconnected physiological compartments: Source Leaf (top), Phloem Stream (middle), and Stolon Tip (bottom), with environmental inputs (day-length and temperature) shown on the left.

(A) Source Leaf—Environmental perception and epigenetic gating. Under inductive short-day (SD) conditions, the repression imposed by Polycomb Repressive Complex 2 (PRC2)-mediated H3K27me3 at the *StSP6A* and *StBEL5* loci is relieved. Concurrently, Trithorax-group (TrxG) complexes deposit permissive H3K4me3 and H3K9Ac marks, remodeling chromatin architecture into an open conformation. This allows transcriptional activation of both the mobile tuberigen protein *StSP6A* and the mobile transcription factor mRNA *StBEL5*. By contrast, under non-inductive long-day (LD) conditions, particularly when combined with heat stress (>25 °C), the repressor *StSP5G* is stabilized and directly suppresses *StSP6A* transcription, effectively blocking the production of systemic signals.

(B) Phloem Stream—Epitranscriptomic filtering and long-distance transport. In the companion cells, full-length *StBEL5* transcripts are selectively marked by N^6^-methyladenosine (m^6^A) at their 3′ untranslated regions (UTRs), catalyzed by the nuclear methyltransferase complex (StMTA/StMTB). These m^6^A modifications serve as physical barcodes recognized by polypyrimidine tract-binding proteins (StPTB1/6), which prevent secondary structure formation and facilitate the active loading of ribonucleoprotein complexes into enucleated sieve elements for systemic translocation. Under elevated temperature, the demethylase StALKBH5 is upregulated and actively removes m^6^A marks from *StBEL5* and *StSP6A* transcripts, leading to their premature endonucleolytic cleavage and degradation within the vascular stream (indicated by red crosses), thus uncoupling the photoperiodic signal from downstream development.

(C) Stolon Tip—Hormonal re-routing and metabolic reprogramming. Upon arrival at the subterranean stolon tip, the mobile StSP6A protein docks with a 14-3-3 scaffolding protein and the bZIP transcription factor StFDL1 to assemble the Tuberization Activation Complex (TAC). The TAC translocates to the nucleus and hyperactivates the gibberellin catabolic gene *StGA2ox1*, generating a local “GA-free window” that arrests longitudinal elongation. Simultaneously, translated StBEL5 upregulates specific kinases that phosphorylate PIN-FORMED auxin efflux carriers (StPIN1/3), triggering their internalization and lateral re-orientation. This re-routes auxin fluxes from the apical pole to the lateral faces, establishing a localized auxin maximum. The TAC also activates cytokinin biosynthesis genes (*StIPT1/3*), generating a cytokinin surge. This synergistic auxin/cytokinin maximum forces a switch from anticlinal to periclinal cell divisions and drives the symplastic-to-apoplastic phloem unloading shift, allowing massive sucrose influx and subsequent trehalose-6-phosphate (T6P)-mediated activation of starch biosynthesis (AGPase, GBSS).

## 2. Scope

This narrative review integrates six interconnected thematic pillars: (i) photoperiodic perception and the core *StSP6A*-mediated tuberigen signaling cascade; (ii) chromatin remodeling and histone methylation gating at key tuberization loci; (iii) mRNA N^6^-methyladenosine (m^6^A) epitranscriptomic modifications that govern phloem mobility and transcript stability; (iv) spatiotemporal hormonal antagonism among gibberellins, auxin, and cytokinins that orchestrates the morphological switch from longitudinal elongation to radial expansion; (v) the symplastic-to-apoplastic phloem unloading shift and trehalose-6-phosphate/SnRK1 metabolic sensing that sustains massive starch deposition; and (vi) translational bottlenecks, including tuberigen thermosensitivity, the genetic complexity of autotetraploidy, and emerging diploid hybrid breeding strategies. The literature search was conducted in PubMed/MEDLINE and Scopus using the following search terms: ‘potato tuberization’, ‘StSP6A’, ‘tuberigen’, ‘m^6^A methylation’, ‘phloem unloading’, ‘gibberellin catabolism’, ‘PIN auxin efflux’, ‘trehalose-6-phosphate’, ‘SnRK1′, and ‘diploid potato breeding’, covering records from 2000 to April 2026. Studies directly investigating molecular, genetic, or physiological mechanisms of tuber development were prioritized, and were supplemented by mechanistic reviews from plant epigenetics, hormone biology, and source–sink physiology. The central hypothesis of this review is that potato tuberization operates as a multi-layered, environmentally gated network in which chromatin and epitranscriptomic modifications act as master filters that integrate systemic photoperiodic cues with local hormonal and metabolic reprogramming, and that targeted manipulation of these nodes—particularly through promoter editing of thermosensitive cis-elements and the transition to diploid hybrid breeding—can effectively decouple tuber initiation from climate-induced suppression, thereby enabling yield stability under projected climate warming conditions. Current evidence robustly supports this framework at the molecular level, yet the translational implementation of these insights into open-field germplasm remains mechanistically and genetically constrained, requiring systematic field-level validation and iterative breeding strategies.

## 3. Environmental Perception and the Epigenetic/Epitranscriptomic Overdrive

The perception of seasonal cues occurs predominantly in the aerial phototropic source leaves [10,11]. The canonical photoperiodic pathway operates through the red/far-red light photoreceptor Phytochrome B (StPHYB) and blue light receptors Cryptochromes [12,13]. Under non-inductive long-day (LD) conditions, active *StPHYB* stabilizes the flowering and tuberization repressor *StCOL1* (CONSTANS-LIKE 1), which directly upregulates the transcription of *StSP5G* [14]. StSP5G acts as a systemic antagonist that suppresses StSP6A transcription [15].

Conversely, under inductive short-day (SD) photoperiods, the day-neutral regulator *StCDF1* (CYCLING DOF FACTOR 1) is stabilized against degradation, directly binding to the promoter of StSP5G to repress its transcription [15]. This relieves the molecular brake on StSP6A expression. Furthermore, systemic amplification loops involving microRNAs, such as *miR172* and *miR156*, act as graft-transmissible signals modulating this cascade [16,17]. However, emerging paradigms demonstrate that this binary transcriptional switch is heavily gated by multi-layered chromatin remodeling and epitranscriptomic modifications [18].

### 3.1. Chromatin Remodeling and Histone Methylation Gating at the StSP6A Locus

The transcriptional transition of *StSP6A* from a silenced state under LD to highly active expression under SD requires a major reconfiguration of the local chromatin landscape [19]. Under non-inductive conditions, the locus of *StSP6A* is tightly encapsulated within a heterochromatic environment maintained by the Polycomb Repressive Complex 2 (PRC2) [19,20]. This complex selectively deposits trimethylation marks on lysine 27 of histone H3 (H3K27me3), a canonical repressive epigenetic modification, across both the promoter region and the first exon of *StSP6A*.

Upon the transition to inductive SD or when plants experience a transient drop in ambient temperature, histone deacetylases (HDACs), including *StHDAC1* and *StHDA6*, are dynamically recruited to the *StSP6A* locus, concurrently causing transcriptional silencing of the repressor *StSP5G* [20]. These deacetylases remove acetyl groups from histone tails, facilitating the eviction of the PRC2 complex [21]. Concurrently, histone methyltransferases belonging to the Trithorax group (TrxG) are activated, leading to a profound enrichment of permissive di- and tri-methylation marks on lysine 4 of histone H3 (H3K4me2/3) and acetylation of lysine 9 of histone H3 (H3K9ac) [22]. This epigenetic remodeling relaxes the local chromatin architecture, rendering the *StSP6A* promoter highly accessible to positive transcriptional activators [23].

A similar mechanism gates the locus of *StBEL5*, a mobile BEL1-like homeodomain transcription factor that acts in tandem with StSP6A [24,25]. Epigenetic assays, including Chromatin Immunoprecipitation sequencing (ChIP-seq), have verified that the promoter of *StBEL5* exhibits an identical H3K27me3-to-H3K4me3 transition upon short-day perception, proving that chromatin accessibility acts as the primary master gatekeeper overseeing the synthesis of long-distance tuberization signals [19].

### 3.2. The Epitranscriptomic Horizon: mRNA m^6^A Methylation Filters

Beyond DNA-level transcriptional gating, the post-transcriptional life cycle of mobile transcripts introduces a critical regulatory layer [26]. Post-transcriptional N^6^-methyladenosine (m^6^A) RNA methylation has recently emerged as a high-fidelity biochemical filter that regulates the stability and translocation efficiency of phloem-mobile mRNAs [27].

Given the well-established role of m^6^A modification in regulating the stability and transport of phloem-mobile mRNAs in plants, it is plausible that the full-length transcripts of *StBEL5* and its heterodimerization partner *StPOTH1* undergo selective m^6^A modifications within their 3′ untranslated regions (3′ UTRs) during the inductive phase of tuberization. In plants, such modifications are catalyzed by a conserved nuclear multi-protein methyltransferase complex comprising MTA and MTB (homologs of mammalian METTL3 and METTL14), which preferentially deposit m^6^A marks near stop codons and within 3′ UTRs [28,29].

*StPTB1* and StPTB6 RNA-binding proteins have been shown to interact with the 3′ UTRs of phloem-mobile *StBEL5* transcripts, enhancing their stability and long-distance transport [30]. Given that m^6^A modifications are prevalent within 3′ UTRs and can modulate RNA fate, it is plausible that the m^6^A status of *StBEL5* transcripts influences their recognition by *StPTB1/6*. Furthermore, this epitranscriptomic tagging may guide the selective sorting and active loading of *StBEL5* transcripts across the plasmodesmata of companion cells into the enucleated sieve elements of the phloem stream—a hypothesis that warrants further investigation within the established phloem-mobile RNA trafficking system [25].

Recent quantitative epitranscriptome sequencing (MeRIP-seq) under stress conditions revealed that when potato plants are exposed to high thermal stress, the expression of the m^6^A demethylase *StALKBH5* (Alkylation Repair Homolog 5) is strongly upregulated [31]. Recent genome-wide identification of the ALKB homolog gene family in potato revealed that *StALKBH* genes are highly expressed in stolons and contain multiple stress-response elements in their promoters, suggesting their potential involvement in the epigenetic regulation of tuberization under stress conditions [31]. However, whether specific *StALKBH* members, such as *StALKBH5*, actively remove m^6^A marks from *StBEL5* and *StSP6A* transcripts to modulate their stability during vascular transport remains an open question that warrants further investigation.” The evolutionary conservation of the core m^6^A regulatory machinery—including methyltransferases (writers), demethylases (erasers), and RNA-binding proteins (readers)—between model plants and potato is summarized in Supplementary Table S1.

**Figure 2:**
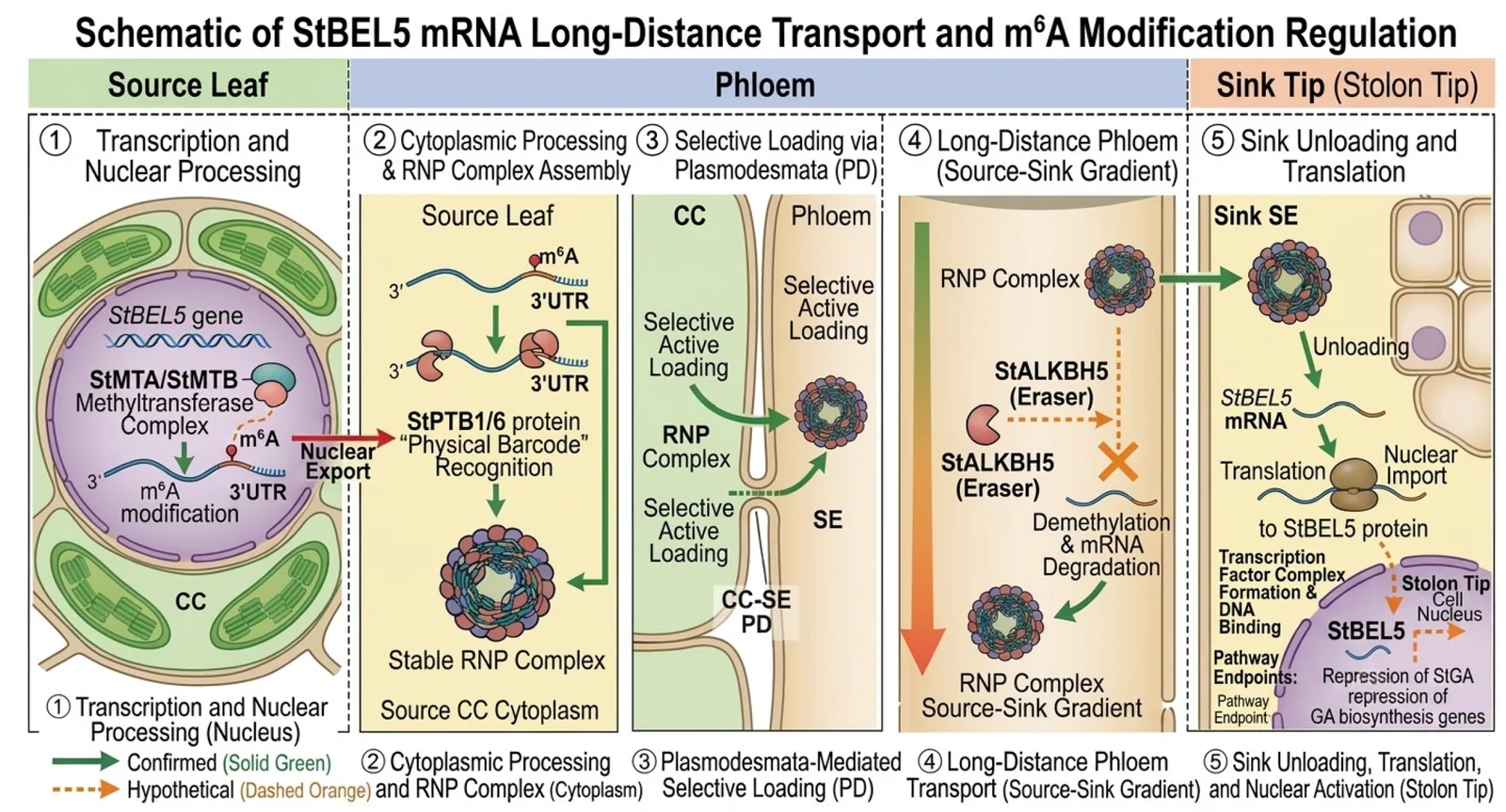
Epitranscriptomic life cycle of the phloem-mobile *StBEL5* transcript during tuberization. The schematic follows the journey of *StBEL5* mRNA from transcription in source leaf companion cell nuclei to translation in stolon tip target cells, highlighting the stepwise roles of m^6^A methylation (*StMTA/StMTB*), m^6^A recognition (*StPTB1/6*), active phloem loading, long-distance translocation, and stress-induced demethylation (*StALKBH5*).

The molecular events of m^6^A deposition, recognition, phloem loading, long-distance translocation, and stress-induced demethylation are synthesized into a sequential model of the *StBEL5* transcript life cycle (Figure 2).

## 4. Hormonal Micro-Environments: Spatiotemporal Antagonism and Morphological Re-Routing

Upon the successful long-distance translocation of the mobile *StSP6A* protein and *StBEL5* mRNA through the phloem into the subterranean stolon, these signals must interface with the local target tissue [4,32]. The primary morphological challenge of tuberization is to halt the rapid longitudinal elongation of the stolon subapical meristem and immediately induce periclinal (radial) cell division and expansion within the subapical tissue [9,33]. This dramatic morphological pivot is executed through a tightly coordinated, self-reinforcing hormonal network that alters local hormone synthesis, catabolism, and polar transport [34].

### 4.1. The Gibberellin (GA) Threshold Shift and Local Catabolism

Active gibberellins, primarily GA_1_ and GA_4_, are the absolute gatekeepers maintaining stolon identity and longitudinal growth [35,36]. High active GA levels stimulate cell elongation by promoting the degradation of DELLA proteins (*StGAI* and *StRGA*), which otherwise repress expansion-related genes [37]. Consequently, establishing a localized “GA-free window” within the subapical zone is a fundamental prerequisite for the onset of tuberization [9,37].

When the mobile *StSP6A* protein enters the companion cells of the stolon tip, it docks with an intracellular 14-3-3 scaffolding protein (specifically St14-3-3-3) and a basic leucine zipper (bZIP) transcription factor designated as *StFDL1* [38,39]. Together, these three components assemble into a highly stable macromolecular structure known as the Tuberization Activation Complex (TAC) [38]. The TAC swiftly translocates into the nucleus of the target cell, where it binds with high affinity to the conserved G-box motifs present within the promoter of the gibberellin catabolic gene *StGA2ox1* [40,41].

The hyper-activation of *StGA2ox1* triggers an immediate, localized surge in active GA degradation, converting GA_1_ and GA_4_ into their biologically inactive 2-beta-hydroxy derivatives [42]. This rapid clearance of local GA permits the stabilization of the potato DELLA protein *StGAI* [37,43]. Stabilized StGAI directly interacts with and de-represses key transcription factors that regulate lateral growth, thereby halting the forward creeping of the stolon and initiating the morphological swelling phase [37].

### 4.2. Polar Auxin Re-Routing and PIN Carrier Re-Orientation

Simultaneously, the local distribution of indole-3-acetic acid (IAA) within the stolon tip undergoes an extensive spatial reconfiguration [44]. In an elongating stolon, auxin is directed primarily toward the tunica layers of the apical meristem to drive longitudinal growth. This directional flux is maintained by the coordinated, basolateral localization of PIN-FORMED auxin efflux carriers, particularly *StPIN1* and *StPIN3* [45,46].

At the onset of swelling, the localized expression of *StBEL5*—translated from the newly arrived mobile mRNA—directly upregulates the transcription of specific protein kinases responsible for phosphorylating PIN proteins [47]. Previous studies have shown that *StPIN2* and *StPIN4* are upregulated prior to stolon swelling, and it has been postulated that StPINs play a role in redistributing auxin in the swelling stolon during early tuber development. Although direct evidence in potato is limited, studies in Arabidopsis have demonstrated that PIN phosphorylation is sufficient to change PIN polarity and redirect auxin fluxes [45,48].

As a consequence of this polar auxin re-routing, a powerful localized auxin maximum is established within the inner cortical layers [46]. This auxin accumulation is associated with the expression of D-type cyclin genes, such as *StCYCD3;2* (Cyclin-D3), which are highly expressed in underground organs and stolons [46]. The resulting change in the cell division plane, from anticlinal to periclinal, drives the radial expansion of the stolon.

### 4.3. Cytokinin Proliferation and Cellular Hyperplasia

Working in absolute synergy with the localized auxin maximum is a synchronized surge in endogenous cytokinins (CKs), specifically trans-zeatin (tZ) [49]. The molecular assembly of the TAC, alongside elevated local auxin levels, directly activates the transcription of *StIPT1* and *StIPT3* (isopentenyltransferase), which encode the rate-limiting enzymes of de novo cytokinin biosynthesis [38,50].

The resulting local enrichment of cytokinins stimulates cell proliferation (hyperplasia) within the medulla and pith tissues of the expanding stolon [50,51]. Cytokinins achieve this by binding to the histidine kinase receptors *StAHK3* and *StAHK4*, which triggers a phosphorylation cascade that activates type-B response regulators (*StARRs*) [52]. These active transcription factors directly stimulate cell wall loosening and expansin genes (*StEXPA1* and *StEXPA4*), allowing the newly divided cells to rapidly expand radially under high internal turgor pressure [53]. This coordinated auxin-cytokinin-driven cellular hyperplasia provides the physical volume required to accommodate massive carbohydrate deposition, a process finely tuned by carotenoid cleavage dioxygenases like *StCCD4* [54] and conserved solanaceous identity modules [55]. Other signals, such as abscisic acid, concurrently coordinate with gibberellins to manage subapical bud dormancy during these architectural switches [56].

**Figure 3:**
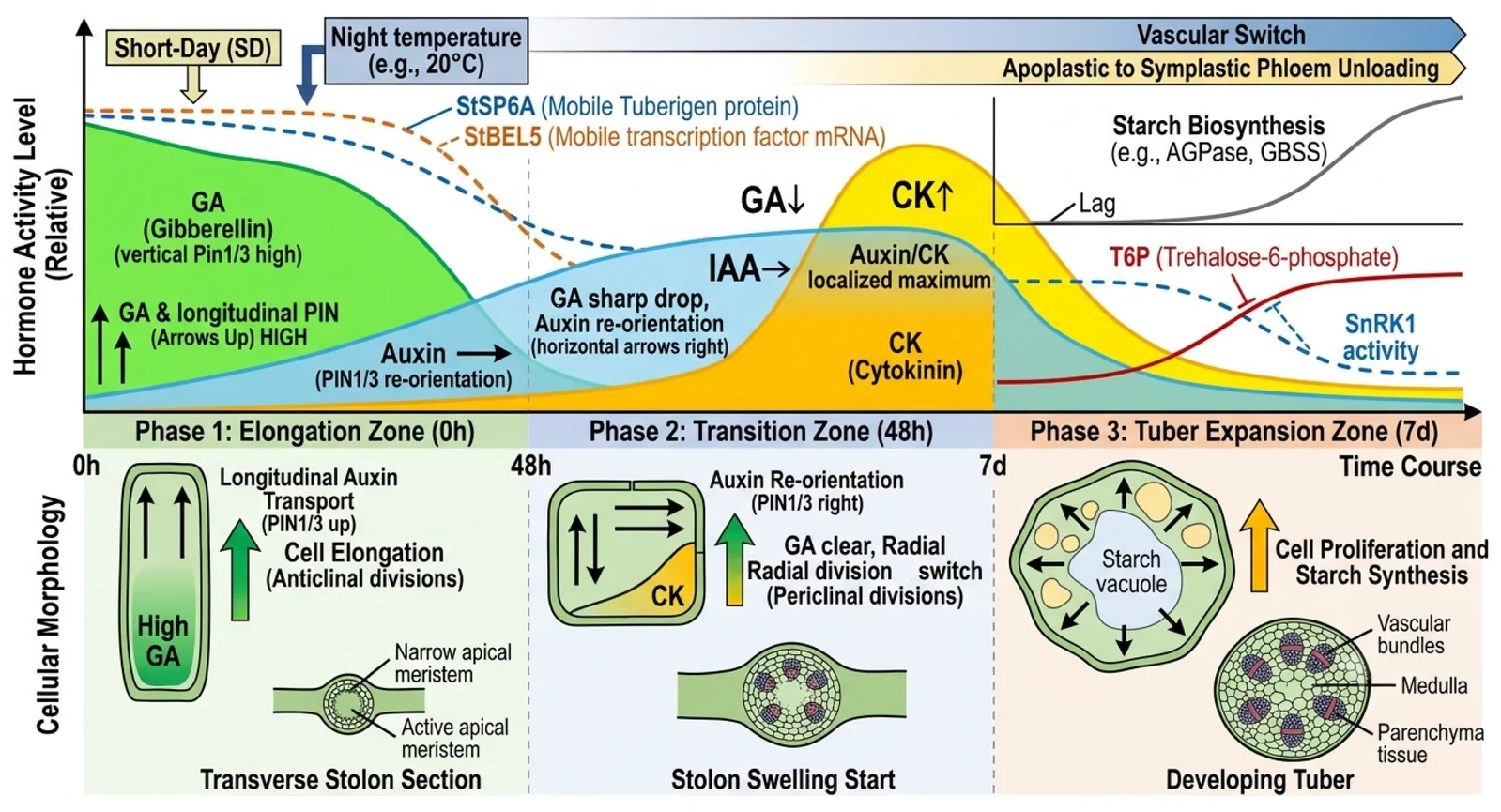
Spatiotemporal phytohormonal dynamics and accompanying cellular morphological transitions during stolon-to-tuber conversion.

The temporal sequence of gibberellin decline, auxin re-orientation, cytokinin surge, and the consequent shift from anticlinal to periclinal cell divisions, as well as the downstream activation of starch biosynthesis, is illustrated in the spatiotemporal model shown in Figure 3.

The figure depicts a temporal continuum (0 h, 48 h, and 7 days post-initiation) divided into three distinct developmental phases, with corresponding transverse stolon cross-sections shown at the bottom.

(A) Phase 1: Elongation Zone (0 h). The elongating stolon is characterized by high bioactive gibberellin (GA) levels and strictly longitudinal (apical) auxin transport, maintained by the basolateral localization of PIN1/3 efflux carriers. This hormonal landscape promotes rapid anticlinal (longitudinal) cell divisions in the subapical meristem, driving active stolon elongation and a narrow, creeping morphology.

(B) Phase 2: Transition Zone (48 h). This marks the critical initiation window. The arrival of the mobile tuberigen signals triggers transcriptional hyperactivation of StGA2ox1, resulting in a steep, localized decline in active GA levels. Concomitantly, PIN1/3 carriers are rapidly internalized and reoriented from the longitudinal to the lateral cell faces. This re-routes auxin fluxes horizontally, generating a strong lateral auxin gradient. Simultaneously, localized cytokinin (CK) biosynthesis surges. The combined GA decline, auxin re-orientation, and CK rise collectively force the division plane of cortical and pith cells to rotate by 90 degrees, shifting from anticlinal to periclinal (radial) divisions, which initiates the visible swelling of the stolon tip.

(C) Phase 3: Tuber Expansion Zone (7 d). The established lateral auxin maximum and sustained high CK levels drive massive cellular hyperplasia (proliferation) in the medulla and storage parenchyma. As sucrose is unloaded into the expanding apoplast, intracellular sucrose accumulates and is sensed by the trehalose-6-phosphate (T6P) pathway. Rising T6P concentrations directly inhibit SnRK1 (SNF1-Related Protein Kinase 1) activity, removing the transcriptional brake on anabolic genes. This metabolic switch depresses ADP-glucose pyrophosphorylase (AGPase) and granule-bound starch synthase (GBSS), leading to massive vacuolar starch accumulation. The bottom cross-sections illustrate the progressive morphological transition from a narrow apical meristem to a radially expanding, starch-filled developing tuber with differentiated vascular bundles and parenchyma tissues.

Abbreviations: GA, gibberellin; CK, cytokinin; IAA, indole‑3‑acetic acid; PIN, PIN‑FORMED auxin transporter; StSP6A, Solanum tuberosum SELF‑PRUNING 6A; StBEL5, Solanum tuberosum BEL‑LIKE 5; T6P, trehalose‑6‑phosphate; T6BP, trehalose‑6‑phosphate phosphatase; AGPase, ADP‑glucose pyrophosphorylase; SPS, sucrose‑phosphate synthase; SPK1, S‑phase kinase‑associated protein 1; SD, short‑day.

## 5. Phloem Unloading and the Symplastic-to-Apoplastic Metabolic Pivot

The morphological expansion of a developing potato tuber must be accompanied by an dramatic increase in carbon allocation [57]. The tuber represents one of the most demanding sink organs in the plant kingdom, demanding up to 80% of the total photoassimilates synthesized by the source leaves [58]. To sustain this massive influx of sucrose without collapsing the vascular transport system, the phloem unloading mechanism within the subterranean stolon undergoes a dynamic, thermodynamic revolution [59].

### 5.1. The Dynamic Shifting of Phloem Unloading Strategies

Within the initial hours of tuber swelling, a sudden surge in sucrose influx can easily create localized osmotic bottlenecks. Rapid accumulation of sucrose in the storage parenchyma cytoplasm leads to a sharp increase in local osmotic potential, which collapses the hydrostatic pressure gradient between the source and sink phloem, rendering long-distance translocation into stagnation [60]. To block this negative feedback mechanism, the expanding tuber tissue synergistically orchestrates a dynamic transition toward apoplastic unloading strategies [59].

The expression of the high-affinity plasma membrane sucrose transporter *StSUT1*, alongside specific cell-wall invertases (*StCWIN1* and *StCWIN3*), is strongly upregulated within the extracellular matrix (apoplast) of the storage parenchyma cells [61]. *StCWIN* irreversibly hydrolyzes the sucrose unloaded into the apoplastic space into glucose and fructose; this biochemical cleavage effectively halves the localized sucrose concentration in the apoplast, thereby sustaining a steep concentration gradient between the sieve element interior and the apoplastic matrix, driving sustained, high-flux passive phloem unloading [62].

Concurrently, sucrose biosynthetic enzymes within the storage parenchyma, such as *StSUSY1* (Sucrose Synthase 1) and *StAGPase* (ADP-glucose pyrophosphorylase), are highly activated during tuber development. The central role of sucrose synthase in carbon partitioning has been well established in model plants, where it contributes to cellulose and starch biosynthesis [63,64]. Sucrose entering the cytoplasm is immediately converted by *StSUSY1* into UDP-glucose and fructose, bypassing the energy-expensive invertase pathways, and is rapidly channeled into amyloplasts for starch polymerization via the post-translational redox modification of AGPase [65]. This highly efficient metabolic conversion ensures that free sucrose concentration within the storage cells remains at an exceptionally low baseline, acting as a permanent “thermodynamic vacuum” that continuously draws photoassimilates out of the phloem stream [7,11].

### 5.2. Trehalose-6-Phosphate (T6P) and SNF1-Related Protein Kinase 1 (SnRK1) Signaling as the Metabolic Gauge

This feedback communication is mediated by the signaling metabolite Trehalose-6-phosphate (T6P), which acts as an ultra-sensitive, real-time proxy for intracellular sucrose concentrations within the expanding storage parenchyma [66].

As sucrose levels surge inside the young tuber, the enzyme *StTPS1* (Trehalose-6-Phosphate Synthase 1) synthesizes an equivalent influx of T6P. Rising intracellular T6P levels bind directly to and inhibit the catalytic activity of *StSnRK1* (SNF1-Related Protein Kinase 1), a central metabolic master regulator that normally promotes catabolism and survival strategies during starvation [67].

The systematic inhibition of StSnRK1 by T6P releases the transcriptional repression on major anabolic and starch biosynthetic genes, including *StGBSS* (Granule-Bound Starch Synthase) and *StSBE* (Starch Branching Enzyme) [66,67]. Concurrently, the T6P-SnRK1 axis generates a vascular feedback signal—involving the minor-vein sucrose transporter *StSUT4*—that travels back to the source leaves [68]. This signal informs the photosynthetic apparatus of the high sink strength below, upregulating leaf carbon export to perfectly match the storage capacity of the expanding underground crop [66,68]. (Table 1)

## 6. High-Information Comprehensive Comparative Matrix

The following comprehensive matrix condenses the molecular, genetic, and transgenic characteristics of the core regulatory components governing the potato tuberization network.

## 7. Translational Trajectories: Bridging the Basic Models to Open-Field Agronomy

While the molecular framework outlined above convincingly explains how photoperiod, hormones, and metabolites coordinate tuber development under controlled conditions, translating this knowledge to open-field agriculture requires confronting additional layers of complexity that laboratory settings systematically exclude. Among these, two stand out as particularly underexplored yet potentially impactful. First, the physical environment of the soil matrix—its compaction, texture, and mechanical impedance—all of which are entirely absent from hydroponic or in vitro systems, yet developing stolons must actively penetrate and swell against this resistance, raising the intriguing but largely unexplored possibility that mechanosensitive signaling pathways may directly modulate tuberization-associated hormonal re-routing. Second, the subterranean stolon tip is not a sterile organ but exists within a dense and dynamic rhizosphere microbial community; whether root- and stolon-associated endophytes or rhizospheric bacteria secrete volatile organic compounds (VOCs) or small-molecule hormonal mimics that bypass, amplify, or suppress the canonical StSP6A-TAC signaling axis is an entirely open question that awaits future investigation. These two largely uncharted dimensions—mechanoperception and rhizobiome interaction—likely constitute significant hidden variables that contribute to the persistent “lab-to-field gap” in potato tuberization research, and they are therefore highlighted in our concluding outlook as priority frontiers for future investigation.

The transition of basic mechanobiology from laboratory benches to commercial open-field production reveals several physiological bottlenecks that compromise agricultural resilience [69]. Addressing these limitations represents the next major milestone for modern crop science.

### 7.1. The Lab-to-Field Gap: Thermosensitivity of the Tuberigen Signal

While laboratory assays conducted under strict climate-controlled growth chambers demonstrate clean, binary responses to day-length changes, open-field performance is increasingly threatened by temperature extremes under global climate change [70]. Ambient temperatures regularly exceeding 25°C cause catastrophic yield penalties across global potato production regions [71].

At the molecular scale, elevated heat exposure overrides the short-day photoperiodic pathway [23]. Heat stress suppresses *StSP6A* expression through temporally distinct regulatory pathways, involving both transcriptional repression and post-transcriptional regulation [72]. Meanwhile, the *miR156/SPL* module has been established as a key photoperiodic regulator of tuberization, with *miR156* overexpression leading to altered plant architecture and reduced tuber yield [17]. Simultaneously, elevated temperatures trigger a systemic surge in *StSP5G* transcription in leaves, completely halting the translocation of the mobile StSP6A protein [72]. This thermal vulnerability causes a complete failure of tuber initiation, resulting in extended stolon branching or aerial heat sprouts instead of market-grade tubers [71,73].

To resolve this Lab-to-Field Gap, current breeding research is pursuing multiple strategies. On the one hand, efforts have focused on identifying genetic determinants of heat tolerance, such as a specific allele of Heat Shock Cognate 70 (*HSc70*) whose temperature-dependent expression improves yield stability under elevated temperatures [74]. On the other hand, understanding the molecular basis of heat-induced tuberization suppression—particularly the downregulation of *StSP6A* expression under high temperature [75]—has provided potential targets for future breeding interventions, including the possible editing of thermosensitive cis-regulatory elements within the *StSP6A* promoter. Decoupling the thermal sensing apparatus from the core tuberigen signaling node represents an essential target for climate-resilient genome editing.

### 7.2. The Polyploid Conundrum and the Diploid Breeding Revolution

The genetic dissection of developmental traits in potato has long been restricted by its inherent genomic complexity of this crop [76]. Modern cultivated potatoes (*Solanum tuberosum* ssp. *tuberosum*) are highly heterozygous autotetraploids (2n = 4x = 48). This arrangement means that every single locus can contain up to four structurally distinct alleles, imposing extreme challenges due to gene dosage effects, multi-allelic interactions, and severe inbreeding depression [77]. Consequently, functional research has heavily relied on self-compatible, homozygous dihaploid reference lines. While useful, these simplified lines fail to mirror the complex allelic subfunctionalization and transcriptional networks present in commercial autotetraploid cultivar.

To break through this genetic bottleneck, the international potato breeding community has initiated a structural transition: the Diploid Hybrid Potato Breeding Strategy, commonly recognized as the “Genome Design of Hybrid Potato” [78,79]. By utilizing gene-editing tools to knock out or knock down the self-incompatibility gene *Sli* (S-locus inhibitor), breeders have successfully generated highly vigorous, self-compatible diploid inbred lines (2n = 2x = 24) [80].

This diploid framework allows researchers to cleanly dissect individual allelic contributions to tuberization [81]. Elite traits—such as optimized *StSUT1* sucrose suction efficiency, heat-tolerant *StSP6A* promoters, and optimal *StGA2ox1* spatial expressions—can now be accurately combined into highly uniform, elite F1 hybrid seeds [81,82]. This transformation replaces the traditional, disease-vulnerable vegetative propagation of seed tubers with clean, high-efficiency sexual hybrid seeds, redefining modern agronomy [78,82].

**Figure 4:**
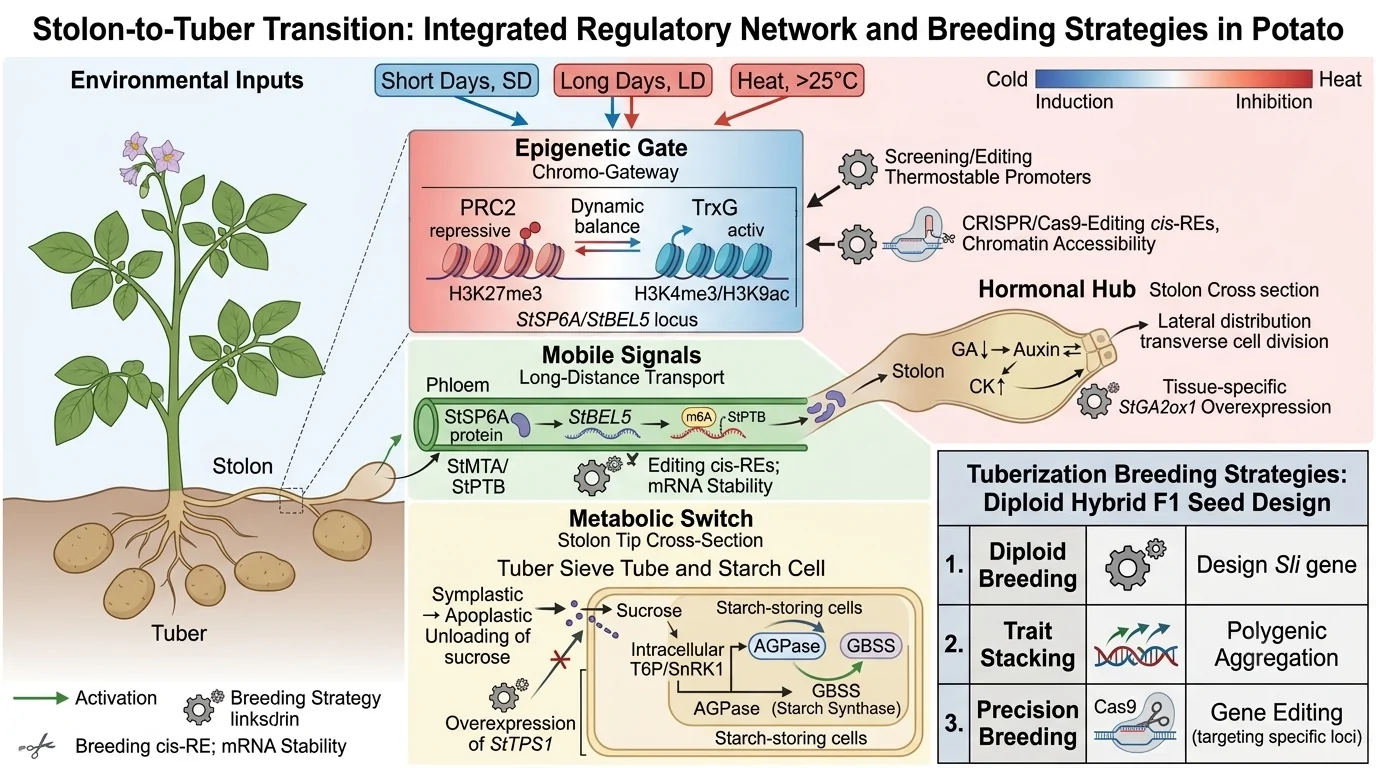
Integrated roadmap from environmental perception to translational breeding strategies for climate-resilient potato. The schematic links six regulatory layers (photoperiodic inputs, chromatin gating, mobile signaling, hormonal reprogramming, metabolic switching, and translational breeding) into a cohesive framework for engineering thermotolerant, day-neutral cultivars.

The six regulatory layers discussed above—photoperiodic perception, chromatin remodeling, mobile signaling, hormonal reprogramming, metabolic switching, and translational breeding—are integrated into a unified framework that links fundamental molecular mechanisms with actionable breeding strategies for climate-resilient potato improvement (Figure 4).

## 8. Conclusions and Outstanding Questions

Our understanding of potato tuberization has successfully transitioned from a flat, linear model to an integrated, multi-layered regulatory architecture. It is now clear that the successful production of an underground potato crop relies on an elegant synchronization that begins with epigenetic modifications in the leaf, proceeds through epitranscriptomic filtering during vascular transport, and culminates in a localized hormonal and metabolic shift at the subterranean stolon meristem.

Despite these substantial molecular clarifications, several critical mechanobiological intersections remain entirely uncharted, representing major opportunities for the future development of the field.

Outstanding Questions for Future Research

Epitranscriptomic Codes: The precise identity and tissue-specific triggers of the specific RNA methyltransferases (“writers”) and demethylases (“erasers”) that add or remove m^6^A chemical barcoding on *StBEL5* and *StSP6A* transcripts under volatile field microclimates remain to be determined.

Mechanosensation of the Soil Matrix: As the stolon subapical meristem initiates periclinal cell division and radial expansion against the physical resistance and density of the surrounding soil matrix, whether mechanosensitive ion channels (e.g., Piezo, OSCA, or Mid1-Complementing Activity homologs) translate soil compaction into localized hormonal re-routing is not yet understood and represents a priority for future research.

The Rhizobiome Link: The extent to which root-and-stolon-associated endophytes or rhizospheric microbial communities secrete volatile organic compounds (VOCs) or small molecule metabolic analogues that actively bypass or amplify the endogenous StSP6A-TAC signaling loop remains to be investigated.

Day-Neutral Thermoresilience Engineering: Can multiplexed CRISPR-Cas9 base-editing of the cis-regulatory sequences of negative effectors (StSP5G and StCOL1) effectively engineer a day-neutral, heat-tolerant elite potato ideotype capable of yielding high-quality starches anywhere across the globe?

## Figures and Tables

**Table 1:** Molecular, genetic, and breeding characteristics of core regulatory components governing potato tuberization.

Regulatory Component	Molecular Identity	Primary Function	Regulatory Mechanism (Activation/Repression)	Transgenic/Mutant Phenotype	Environmental Sensitivity	Breeding/Target Potential
*StSP6A*	PEBP family protein	Mobile “tuberigen”; initiates TAC assembly at stolon tip	Transcriptional activation under SD via relief of *StSP5G* repression; protein stabilized by 14-3-3	Overexpression → tuberization under LD; RNAi → no tubers	Strongly suppressed by heat (>25 °C) via *StSP5G* upregulation and protein degradation	Promoter editing for heat-tolerant alleles; top candidate for gene editing
*StBEL5*	BEL1-like homeodomain transcription factor	Mobile mRNA; coordinates auxin/PIN re-orientation and cell division plane shift	m^6^A methylation at 3‘UTR enables phloem loading; transcribed synergistically with *StSP6A*	Overexpression → increased tuber yield and stolon branching; KD → reduced swelling	Sensitive to heat via *StALKBH5*-mediated m^6^A demethylation and mRNA decay	mRNA stability engineering; upregulation of methyltransferase complex
*StSP5G*	PEBP family protein (antagonist)	Systemic florigen/tuberigen inhibitor; represses *StSP6A* transcription	Directly induced by *StCOL1* under LD; repressed by *StCDF1* under SD	Overexpression → complete suppression of tuberization; loss-of-function → day-neutrality	Upregulated by high temperature, overriding SD signals	CRISPR knockout for day-neutral, heat-resilient ideotypes
*StGA2ox1*	Gibberellin 2-oxidase	GA catabolic enzyme; creates “GA-free window” at stolon subapical zone	Direct target of TAC (*StSP6A*-14-3-3-FDL1); transcriptional hyperactivation	Overexpression → ectopic tuberization on elongating stolons; KO → delayed initiation	Indirectly affected by temperature via *StSP6A* abundance	Tissue-specific (stolon tip) overexpression under native promoter
*StPIN1/3*	PIN-FORMED auxin efflux carriers	Polar auxin transport; reoriented from longitudinal to lateral faces	Phosphorylated and internalized/re-localized by *StBEL5*-upregulated kinases	Mis-expression → altered stolon curvature and swelling asymmetry	Likely affected by soil mechanical impedance	Chemical or genetic modulation of phosphorylation status
*StSUT1/StSUT4*	Sucrose transporters	*StSUT1*: phloem loading; *StSUT4*: vascular feedback signaling	*StSUT4* senses *T6P/SnRK1* status to adjust leaf export	*StSUT1* suppression → stunted tuber fill; *StSUT4* loss → altered allocation	Sensitive to sink-strength feedback; *T6P* upregulation enhances activity	Overexpression of *StSUT1* for super-sink cultivars
*T6P/SnRK1*	Trehalose-6-phosphate; SNF1-related kinase	Metabolic gauge; T6P inhibits SnRK1 to de-repress starch synthesis	*T6P* synthesized by *StTPS1* in proportion to sucrose influx; inhibits SnRK1 kinase activity	Altered *T6P* levels → drastic changes in starch content and tuber size	Directly responds to fluctuating sucrose and oxygen levels	Targeted increase of *T6P* via *StTPS1* overexpression for higher yield

The table summarizes the key regulatory components involved in potato tuberization, organized by their molecular identity, primary function, regulatory mechanism (activation/repression), transgenic or mutant phenotype, environmental sensitivity, and breeding/target potential. Abbreviations not defined in the main text are as follows: KD, knockdown; KO, knockout; OE, overexpression; RNAi, RNA interference; TAC, Tuberization Activation Complex; *T6P*, trehalose-6-phosphate; *SnRK1*, *SNF1*-related protein kinase 1. This comparative matrix is intended to provide a rapid overview of the genetic nodes that are most amenable to targeted modification for breeding climate-resilient and high-yielding potato cultivar.

## Data Availability

No new data were created or analyzed in this study. Data sharing is not applicable to this article.

## Supplementary Materials

The following supporting information can be downloaded at: https://www.mdpi.com/article/10.3390/ijms27167149/s1.

## Abbreviations

The following abbreviations are used in this manuscript:

AGPase	ADP-glucose pyrophosphorylase
ChIP-seq	Chromatin Immunoprecipitation sequencing
CK	Cytokinin
CRISPR	Clustered Regularly Interspaced Short Palindromic Repeats
CWIN	Cell-wall invertase
GA	Gibberellin
GBSS	Granule-bound starch synthase
HDAC	Histone deacetylase
IAA	Indole-3-acetic acid
LD	Long-day
m^6^A	N^6^-methyladenosine
MeRIP-seq	Methylated RNA Immunoprecipitation sequencing
PIN	PIN-FORMED (auxin efflux carrier)
PRC2	Polycomb Repressive Complex 2
RNAi	RNA interference
SBE	Starch branching enzyme
SD	Short-day
SnRK1	SNF1-related protein kinase 1
T6P	Trehalose-6-phosphate
TAC	Tuberization Activation Complex
TrxG	Trithorax group
UTR	Untranslated region
VOC	Volatile organic compound

## References

[1] Birch P.R.J., Bryan G., Fenton B., Gilroy E.M., Hein I., Jones J.T., Prashar A., Taylor M.A., Torrance L., Toth I.K. (2012). Crops that feed the world 8: Potato: Are the trends of increased global production sustainable?. Food Secur..

[2] Xu X., Vreugdenhil D., Lammeren A.A.M. (1998). Cell division and cell enlargement during potato tuber formation. J. Exp. Bot..

[3] Jackson S.D., Heyer A., Dietze J. (2010). Phytochrome B mediates the photoperiodic control of tuber formation in potato. Plant J..

[4] Abelenda J.A., Bergonzi S., Oortwijn M., Sonnewald S., Du M., Visser R.G.F., Sonnewald U., Bachem C.W.B. (2019). Source-sink regulation is mediated by interaction of an FT homolog with a SWEET protein in potato. Curr. Biol..

[5] Navarro C., Abelenda J.A., Cruz-Oró E., Cuéllar C.A., Tamaki S., Silva J., Shimamoto K., Prat S. (2011). Control of flowering and storage organ formation in potato by FLOWERING LOCUS T. Nature.

[6] Abelenda J.A., Navarro C., Prat S. (2014). Flowering and tuberization: A tale of two nightshades. Trends Plant Sci..

[7] Fernie A.R., Willmitzer L. (2001). Molecular and biochemical triggers of potato tuber development. Plant Physiol..

[8] Kondhare K., Kumar A., Patil N., Malankar N., Saha K., Banerjee A. (2021). Development of aerial and belowground tubers in potato is governed by photoperiod and epigenetic mechanism. Plant Physiol..

[9] Kondhare K., Natarajan B., Banerjee A. (2020). Molecular signals that govern tuber development in potato. Int. J. Dev. Biol..

[10] Dutta M., Mali S., Raturi V., Zinta G. (2024). Transcriptional and post-transcriptional regulation of tuberization in potato (*Solanum tuberosum* L.). J. Plant Growth Regul..

[11] Singh P., Arif Y., Siddiqui H., Upadhyaya C.P., Pichtel J., Hayat S. (2023). Critical factors responsible for potato tuberization. Bot. Rev..

[12] Martínez-García J.F., Virgós-Soler A., Prat S. (2002). Control of photoperiod-regulated tuberization in potato by the *Arabidopsis* flowering-time gene *CONSTANS*. Proc. Natl. Acad. Sci. USA.

[13] Kasprzewska A., Basińska-Barczak A. (2025). Molecular mechanisms of dual potato reproduction. Physiol. Plant..

[14] Abelenda J.A., Cruz-Oró E., Franco-Zorrilla J.M., Prat S. (2016). Potato StCONSTANS-like1 suppresses storage organ formation by directly activating the FT-like StSP5G repressor. Curr. Biol..

[15] Kloosterman B., Abelenda J.A., Gomez M.D.M.C., Oortwijn M., de Boer J.M., Kowitwanich K., Horvath B.M., van Eck H.J., Smaczniak C., Prat S. (2013). Naturally occurring allele diversity allows potato cultivation in northern latitudes. Nature.

[16] Martin A., Adam H.L.N., Diaz-Mendoza M., Żurczak M., González-Schain N., Suárez-López P. (2009). Graft-transmissible induction of potato tuberization by the microRNA miR172. Development.

[17] Bhogale S., Mahajan A.S., Natarajan B., Rajabhoj M., Thulasiram H.V., Banerjee A.K. (2014). MicroRNA156: A potential graft-transmissible microRNA that modulates plant architecture and tuberization in *Solanum tuberosum* ssp. *andigena*. Plant Physiol..

[18] Zhang Z., Zhou J., Zhao Y., Zhao X., Liu J., Liu J., Zhang J., Song B., Zhang H. (2024). StMYB113 promotes anthocyanin biosynthesis in potato (*Solanum tuberosum* L.) Desiree tubers. Potato Res..

[19] Kumar A., Kondhare K.R., Malankar N.N., Banerjee A.K. (2021). The Polycomb group methyltransferase StE(z)2 and deposition of H3K27me3 and H3K4me3 regulate the expression of tuberization genes in potato. J. Exp. Bot..

[20] Kumar A., Kondhare K.R., Vetal P.V., Banerjee A.K. (2020). PcG proteins MSI1 and BMI1 function upstream of miR156 to regulate aerial tuber formation in potato. Plant Physiol..

[21] Wang S., Fairall L., Pham T.K., Ragan T.J., Vashi D., Collins M.O., Dominguez C., Schwabe J.W.R. (2023). A potential histone-chaperone activity for the MIER1 histone deacetylase complex. Nucleic Acids Res..

[22] Singh S., Mantri M.P., Khan M.I., Banerjee A.K. (2025). A comprehensive analysis to speculate the role of trithorax genes in photoperiod-mediated tuberization in potato and comparison with storage root crops. BMC Plant Biol..

[23] Morris W.L., Ducreux L.J.M., Morris J., Campbell R., Usman M., Hedley P.E., Prat S., Taylor M.A. (2019). Identification of timing of CAB expression1 as a temperature-sensitive negative regulator of tuberization in potato. J. Exp. Bot..

[24] Banerjee A.K., Chatterjee M., Yu Y., Suh S.G., Miller W.A., Hannapel D.J. (2006). Dynamics of a mobile RNA of potato involved in a long-distance signaling pathway. Plant Cell.

[25] Hannapel D.J., Banerjee A.K. (2017). Multiple mobile mRNA signals regulate tuber development in potato. Plants.

[26] Mahajan A., Bhogale S., Kang I.H., Hannapel D.J., Banerjee A.K. (2012). The mRNA of a Knotted1-like transcription factor of potato is phloem mobile. Plant Mol. Biol..

[27] Li Q., Wang Y., Gao Y., Wang Q., Niu Z., Nan X., Fan G., Sheng W. (2025). Comparative transcriptome analysis of genes related to tuberization in late and early maturing potato (*Solanum tuberosum* L.) cultivars. Sci. Rep..

[28] Zhong S., Li H., Bodi Z., Button J., Vespa L., Herzog M., Fray R.G. (2008). MTA is an Arabidopsis messenger RNA adenosine methylase and interacts with a homolog of a sex-specific splicing factor. Plant Cell.

[29] Tang J., Chen S., Jia G. (2023). Detection, regulation, and functions of RNA N6-methyladenosine modification in plants. Plant Commun..

[30] Cho S.K., Sharma P., Butler N.M., Kang I.H., Shah S., Rao A.G., Hannapel D.J. (2015). Polypyrimidine tract-binding proteins of potato mediate tuberization through an interaction with StBEL5 RNA. J. Exp. Bot..

[31] Li Y., Dong X., Ma J., Sui C., Jian H., Lv D. (2024). Genome-wide identification and expression analysis of the ALKB homolog gene family in potato (*Solanum tuberosum* L.). Int. J. Mol. Sci..

[32] Bao X., Zhu Y., Li G., Liu L. (2025). Regulation of storage organ formation by long-distance tuberigen signals in potato. Hortic. Res..

[33] Zhang M., Jian H., Shang L., Wang K., Wen S., Li Z., Liu R., Jia L., Huang Z., Lyu D. (2024). Transcriptome analysis reveals novel genes potentially involved in tuberization in potato. Plants.

[34] Saidi A., Hajibarat Z. (2021). Phytohormones: Plant switchers in developmental and growth stages in potato. J. Genet. Eng. Biotechnol..

[35] Kondhare K.R., Patil A.B., Giri A.P. (2021). Auxin: An emerging regulator of tuber and storage root development. Plant Sci..

[36] Kaur Y., Das N. (2024). Gibberellin 2-oxidases in potato (*Solanum tuberosum* L.): Cloning, characterization, in silico analysis and molecular docking. Mol. Biotechnol..

[37] Blanco-Tourinan N., Serrano-Mislata A., Alabadi D. (2020). Regulation of DELLA proteins by post-translational modifications. Plant Cell Physiol..

[38] Teo C.J., Takahashi K., Shimizu K., Shimamoto K., Taoka K.I. (2017). Potato tuber induction is regulated by interactions between components of a tuberigen complex. Plant Cell Physiol..

[39] Harada K.I., Furuita K., Yamashita E., Taoka K.I., Tsuji H., Fujiwara T., Nakagawa A., Kojima C. (2022). Crystal structure of potato 14-3-3 protein St14f revealed the importance of helix I in StFDL1 recognition. Sci. Rep..

[40] Jing S., Sun X., Yu L., Wang E., Cheng Z., Liu H., Jiang P., Qin J., Begum S., Song B. (2022). Transcription factor StABI5-like 1 binding to the FLOWERING LOCUS T homologs promotes early maturity in potato. Plant Physiol..

[41] Sun X., Wang E., Yu L., Liu S., Liu T., Qin J., Jiang P., He S., Cai X., Jing S. (2024). TCP transcription factor StAST1 represses potato tuberization by regulating tuberigen complex activity. Plant Physiol..

[42] Kloosterman B., Navarro C., Bijsterbosch G., Lange T., Prat S., Visser R.G.F., Bachem C.W.B. (2007). StGA2ox1 is induced prior to stolon swelling and controls GA levels during potato tuber development. Plant J..

[43] Sarwar R., Zhu K.M., Jiang T., Ding P., Gao Y., Tan X.L. (2023). DELLAs directed gibberellins responses orchestrate crop development: A brief review. Crop Sci..

[44] Kolachevskaya O.O., Sergeeva L.I., Flokova K., Getman I.A., Lomin S.N., Alekseeva V.V., Rukavtsova E.B., Buryanov Y.I., Romanov G.A. (2017). Auxin synthesis gene tms1 driven by tuber-specific promoter alters hormonal status of transgenic potato plants and their responses to exogenous phytohormones. Plant Cell Rep..

[45] Roumeliotis E., Kloosterman B., Oortwijn M., Visser R.G.F., Bachem C.W.B. (2013). The PIN family of proteins in potato and their putative role in tuberization. Front. Plant Sci..

[46] Roumeliotis E., Kloosterman B., Oortwijn M., Kohlen W., Bouwmeester H.J., Visser R.G.F., Bachem C.W.B. (2012). The effects of auxin and strigolactones on tuber initiation and stolon architecture in potato. J. Exp. Bot..

[47] Sharma P., Lin T., Hannapel D.J. (2016). Targets of the StBEL5 transcription factor include the FT ortholog StSP6A. Plant Physiol..

[48] Zhang J., Nodzynski T., Pencik A., Rolcik J., Friml J. (2010). PIN phosphorylation is sufficient to mediate PIN polarity and direct auxin transport. Proc. Natl. Acad. Sci. USA.

[49] Kolachevskaya O.O., Lomin S.N., Arkhipov D.V., Romanov G.A. (2019). Auxins in potato: Molecular aspects and emerging roles in tuber formation and stress resistance. Plant Cell Rep..

[50] Lomin S., Myakushina Y., Kolachevskaya O., Getman I., Savelieva E., Arkhipov D., Deigraf S., Romanov G. (2020). Global view on the cytokinin regulatory system in potato. Front. Plant Sci..

[51] Cheng L., Wang D., Wang Y., Xue H., Zhang F. (2019). An integrative overview of physiological and proteomic changes of cytokinin-induced potato (*Solanum tuberosum* L.) tuber development in vitro. Physiol. Plant..

[52] Lomin S.N., Myakushina Y.A., Kolachevskaya O.O., Getman I.A., Arkhipov D.V., Savelieva E.M., Osolodkin D.I., Romanov G.A. (2018). Cytokinin perception in potato: New features of canonical players. J. Exp. Bot..

[53] Chen Y., Zhang B., Li C., Lei C., Kong C., Yang Y., Gong M. (2019). A comprehensive expression analysis of the expansin gene family in potato (*Solanum tuberosum*) discloses stress-responsive expansin-like B genes for drought and heat tolerances. PLoS ONE.

[54] Campbell R., Ducreux L.J., Morris W.L., Morris J.A., Suttle J.C., Ramsay G., Bryan G.J., Hedley P.E., Taylor M.A. (2010). The metabolic and developmental roles of carotenoid cleavage dioxygenase4 from potato. Plant Physiol..

[55] Eviatar-Ribak T., Shalit-Kaneh A., Chappell-Maor L., Amsellem Z., Eshed Y., Lifschitz E. (2013). A cytokinin-activating enzyme promotes tuber formation in tomato. Curr. Biol..

[56] Mbugua J., Kabira J., Shimelis H., Melis R. (2014). Regulation of potato tuber dormancy: A review. Aust. J. Crop Sci..

[57] Sonnewald U., Kossmann J. (2013). Starches—From current models to genetic engineering. Plant Biotechnol. J..

[58] Halford N.G. (1999). Metabolic signalling and the partitioning of resources in plant storage organs. J. Agric. Sci..

[59] Viola R., Roberts A.G., Haupt S., Gazzani S., Hancock R.D., Marmiroli N., Machray G.C., Oparka K.J. (2001). Tuberization in potato involves a switch from apoplastic to symplastic phloem unloading. Plant Cell.

[60] Golovko T.K., Tabalenkova G.N. (2019). Source-sink relationships in potato plants. Russ. J. Plant Physiol..

[61] Roitsch T., Gonzalez M.C. (2004). Function and regulation of plant invertases: Sweet sensations. Trends Plant Sci..

[62] Hajirezaei M.R., Takahata Y., Trethewey R.N., Willmitzer L., Sonnewald U. (2000). Impact of elevated cytosolic and apoplastic invertase activity on carbon metabolism during potato tuber development. J. Exp. Bot..

[63] Baroja-Fernandez E., Munoz F.J., Li J., Bahaji A., Almagro G., Montero M., Etxeberria E., Hidalgo M., Sesma M.T., Pozueta-Romero J. (2012). Sucrose synthase activity in the *sus1/sus2/sus3/sus4* Arabidopsis mutant is sufficient to support normal cellulose and starch production. Proc. Natl. Acad. Sci. USA.

[64] Geigenberger P. (2003). Regulation of sucrose to starch conversion in growing potato tubers. J. Exp. Bot..

[65] Tiessen A., Hendriks J.H.M., Stitt M., Branscheid A., Gibon Y., Farre E.M., Geigenberger P. (2002). Starch synthesis in potato tubers is regulated by post-translational redox modification of ADP-glucose pyrophosphorylase: A novel regulatory mechanism linking starch synthesis to the sucrose supply. Plant Cell.

[66] Fichtner F., Lunn J.E. (2021). The role of trehalose 6-phosphate (Tre6P) in plant metabolism and development. Annu. Rev. Plant Biol..

[67] Debast S., Nunes-Nesi A., Hajirezaei M.R., Hofmann J., Sonnewald U., Fernie A.R., Bornke F. (2011). Altering trehalose-6-phosphate content in transgenic potato tubers affects tuber growth and alters responsiveness to hormones during sprouting. Plant Physiol..

[68] Chincinska I.A., Liesche J., Krugel U., Michalska J., Geigenberger P., Grimm B., Kuhn C. (2008). Sucrose transporter StSUT4 from potato affects flowering, tuberization, and shade avoidance response. Plant Physiol..

[69] Dahal K., Li X.Q., Tai H., Creelman A., Bizimungu B. (2019). Improving potato stress tolerance and tuber yield under a climate change scenario—A current overview. Front. Plant Sci..

[70] Rykaczewska K. (2015). The effect of high temperature occurring in subsequent stages of plant development on potato yield and tuber physiological defects. Am. J. Potato Res..

[71] Hancock R.D., Morris W.L., Ducreux L.J.M., Morris J.A., Usman M., Verrall S.R., Fuller J., Simpson C.G., Zhang R., Hedley P.E. (2014). Physiological, biochemical and molecular responses of the potato (*Solanum tuberosum* L.) plant to moderately elevated temperature. Plant Cell Environ..

[72] Park J.S., Park S.J., Kwon S.Y., Shin A.Y., Moon K.B., Park J.M., Cho H.S., Park S.U., Jeon J.H., Kim H.S. (2022). Temporally distinct regulatory pathways coordinate thermo-responsive storage organ formation in potato. Cell Rep..

[73] Reynolds M.P., Ewing E.E. (1989). Effects of high air and soil temperature stress on growth and tuberization in Solanum tuberosum. Ann. Bot..

[74] Trapero-Mozos A., Morris W.L., Ducreux L.J.M., McLean K., Stephens J., Torrance L., Bryan G.J., Hancock R.D., Taylor M.A. (2018). Engineering heat tolerance in potato by temperature-dependent expression of a specific allele of heat-shock cognate 70. Plant Biotechnol. J..

[75] Hastilestari B.R., Lorenz J., Reid S., Hofmann J., Pscheidt D., Sonnewald U., Sonnewald S. (2018). Deciphering source and sink responses of potato plants (*Solanum tuberosum* L.) to elevated temperatures. Plant Cell Environ..

[76] Kyriakidou M., Anglin N.L., Ellis D., Tai H.H., Stromvik M.V. (2020). Genome assembly of six polyploid potato genomes. Sci. Data.

[77] Kyriakidou M., Achakkagari S.R., Galvez Lopez J.H., Zhu X., Tang C.Y., Tai H.H., Anglin N.L., Ellis D., Stromvik M.V. (2020). Structural genome analysis in cultivated potato taxa. Theor. Appl. Genet..

[78] Jansky S.H., Charkowski A.O., Douches D.S., Gusmini G., Richael C., Bethke P.C., Spooner D.M., Novy R.G., De Jong H., De Jong W.S. (2016). Reinventing potato as a diploid inbred line–based crop. Crop Sci..

[79] Zhang C., Yang Z., Tang D., Zhu Y., Wang P., Li D., Zhu G., Xiong X., Shang Y., Li C. (2021). Genome design of hybrid potato. Cell.

[80] Eggers E.J., van der Burgt A., van Heusden S.A.W., de Vries M.E., Visser R.G.F., Bachem C.W.B., Lindhout P. (2021). Neofunctionalisation of the Sli gene leads to self-compatibility and facilitates precision breeding in potato. Nat. Commun..

[81] Tang D., Jia Y., Zhang J., Li H., Cheng L., Wang P., Bao Z., Liu Z., Feng S., Zhu Y. (2022). Genome evolution and diversity of wild and cultivated potatoes. Nature.

[82] Stockem J.E., Bus M.D., de Vries M.E., Struik P.C. (2024). Opening eyes on seedling tuber quality in potato: Size matters. Potato Res..

