# Supplementary material for: Unraveling the Intertwined Networks of Potato Tuberization: From Epitranscriptomic Signaling to Metabolic Reprogramming
Source: Int J Mol Sci. 2026 Aug 10;27(16):7149. doi: 10.3390/ijms27167149 (PMC13513807; doi:10.3390/ijms27167149)
Supplement: Supplementary file 1 [file ijms-27-07149-s001.zip › ijms-4464189-supplementary.pdf]

Supplementary Table S1. Evolutionary conservation of m^6A regulatory machinery between model plants and potato.

Regulator type	Arabidopsis thaliana/mammalian homologs	Solanum tuberosum homologs	Validated functions in Solanum tuberosum
Writer (Methyltransferase)	METTL3 (MTA)	StMTA	Catalytic subunit of m^6A methyltransferase
Writer	METTL14 (MTB)	StMTB	Forms a heterodimer with StMTA
Writer	WTAP (FIP37)	StFIP37	Regulates m^6A deposition
Eraser (Demethylase)	ALKBH5 (in plants)	StALKBH5	Upregulated under heat stress (putative)
Reader (Recognition protein)	YTHDF (ECT)	StECT	Recognizes m^6A and regulates RNA stability
Reader-like	PTB (in animals)	StPTB1/6	Enhances stability by binding to the 3'UTR of StBEL5

Supplementary Table S1. The table summarizes the core components of the m^6A epitranscriptomic apparatus—including methyltransferases (writers), demethylases (erasers), and RNA-binding proteins (readers/reader-like proteins)—identified in Arabidopsis thaliana/mammals and their corresponding homologs in potato (Solanum tuberosum). Functional characterization in potato is indicated where available. This comparison highlights the conserved nature of the m^6A modification system across plant species and provides a framework for investigating its roles in potato tuberization. Abbreviations: MTA, mRNA Methyltransferase A; MTB, mRNA Methyltransferase B; FIP37, FKBP12 Interacting Protein 37; WTAP, Wilms Tumor 1-Associating Protein; ALKBH5, Alkylation Repair Homolog 5; YTHDF, YTH Domain-Containing Family Protein; ECT, Evolutionarily Conserved C-Terminal Region; PTB, Polypyrimidine Tract-Binding Protein. References for validated functions: StMTA/StMTB[26]; StFIP37[27]; StALKBH5[29]; StPTB1/6[28].
